# 5-AzaCytidine Promotes Somatic Embryogenesis of *Taxodium* Hybrid ‘Zhongshanshan’ by Regulating Redox Homeostasis

**DOI:** 10.3390/plants14091354

**Published:** 2025-04-30

**Authors:** Guoying Yuan, Dan Wang, Chaoguang Yu, Jianfeng Hua, Yunlong Yin, Tingting Chen

**Affiliations:** 1 College of Forestry and Grassland and College of Soil and Water, Nanjing Forestry University, Nanjing 210037, China; yuanyy0423@163.com; 2 Institute of Botany, Jiangsu Province and Chinese Academy of Sciences (Nanjing Botanical Garden Mem. Sun Yat-Sen), No. 1, Qianhu Village, Zhongshan Gate, Nanjing 210014, China; wdan0423@163.com (D.W.); yuchaoguang168@cnbg.net (C.Y.); jfhua@cnbg.net (J.H.); 3 Jiangsu Key Laboratory for Conservation and Utilization of Plant Resources, Nanjing 210014, China

**Keywords:** 5-azaC, action time, maturation rate, ROS, somatic embryos, *Taxodium*

## Abstract

DNA methylation plays a crucial role in regulating the developmental processes of plants. Particularly, it is closely associated with the development of embryogenic cells (EC) and somatic embryos (SE). In this study, we investigated the effects of 5-azaCytidine (5-azaC) treatment on somatic embryogenesis proliferation and maturation of *Taxodium* hybrid ‘zhongshanshan’. The results showed that the callus proliferation was inhibited when the concentration of 5-azaC exceeded 30 μM, while treatment with 5 μM 5-azaC improved the maturation rate and expedited the process of SE formation. It was also noted that 5-azaC influenced somatic embryogenesis during the second week of embryo induction, substantially enhancing the maturation rate of somatic embryos and the germination rate of *Taxodium* hybrid ‘zhongshanshan’. Furthermore, the analysis revealed that treatment with 5-azaC resulted in elevated levels of H_2_O_2_, SOD, POD, and AsA during the cotyledonary embryo period in *Taxodium* hybrid ‘zhongshanshan’, indicating its potential to promote somatic embryogenesis by regulating redox homeostasis. This study concluded that 5-azaC could improve the efficiency of somatic embryogenesis in *Taxodium* hybrid ‘zhongshanshan’, as well as provide a solid foundation for investigating the effects of 5-azaC on somatic embryogenesis in other conifer species.

## 1. Introduction

*Taxodium* hybrid ‘zhongshanshan’ denotes exceptional intraspecific hybrids within the Taxodium genus, which belongs to *Cupressaceae*, and is formed by hybridizing *Taxodium distichum* (L.) Rich, *Taxodium ascendens* Brongn, and *Taxodium mucronatum* Ten [1]. Over years of planting experiments, these hybrids exhibited superior traits including rapid growth, saline-alkali resistance, moisture tolerance, and drought tolerance [2]. However, traditional breeding methods like crossbreeding and cutting propagation suffer from drawbacks such as long cycles, low efficiency, and restricted reproductive factors. Therefore, establishing an effective in vitro regeneration technology system is essential for biological breeding. Nevertheless, challenges in somatic embryogenesis of *Taxodium* hybrid ‘zhongshanshan’, such as a low induction rate of somatic embryogenesis, a high occurrence of abnormal embryos, and poor germination rates, have hindered its practical application. Consequently, research efforts focused on enhancing the efficiency of in vitro regeneration and understanding its mechanisms are essential for advancing the field of biological breeding for *Taxodium* hybrid ‘zhongshanshan’.

Somatic embryogenesis is one of the main manifestations of cell totipotency, a process in which any somatic cell has the capacity to produce a whole plant without gamete fusion [3]. It is an important biotechnology due to its advantages of gene stability, reproducibility, and high efficiency. Various hormones, stresses, transcription factors, as well as complex epigenetic modifications play crucial roles in influencing somatic embryogenesis [4,5,6]. Epigenetic modifications primarily comprise DNA methylation and histone acetylation [7], which have been suggested to significantly impact somatic embryogenesis by modulating gene expression patterns. DNA methylation is a well-characterized epigenetic marker [8] that can dynamically change during plant cell differentiation and proliferation, consequently influencing gene expression [9].

In plant growth, DNA methylation plays a crucial role in regulating gene expression, which primarily influences DNA structure, DNA–protein interactions, chromatin structure, and histone modifications. The compound 5-azaC (5-azaCytidine), an analog of cytosine, acts as a DNA methyltransferase inhibitor (DNMT inhibitor) [10]. Its mechanism involves incorporating DNA/RNA, targeting DNA methyltransferase, and decreasing its activity within cells to inhibit methylation [11]. Furthermore, as a methylation inhibitor, 5-azaC efficiently modulates methylation levels during plant development and regulates the expression of stress-related genes, especially under fluctuating environmental conditions that induce plant stress [12,13]. Some research has emphasized the significant role of 5-azaC in reducing DNA methylation levels during plant embryo development [11,14]. When utilized as a demethylating agent in various plant regeneration systems, the impact of 5-azaC on plant growth depends on variables such as concentration and duration of treatment [15,16]. Modifying or reducing the induced DNA methylation level following 5-azaC treatment can influence embryogenic capacity.

Somatic embryogenesis induction is a complex process influenced by numerous factors. One of the key elements affecting somatic embryogenesis is oxidative stress, which is characterized by the accumulation of varying levels of reactive oxygen species (ROS) [17,18]. ROS can also act as signaling molecules to stimulate cellular adaptation, thereby influencing somatic embryogenesis [19]. High concentrations of ROS are toxic and can induce oxidative damage. However, an appropriate level of ROS provides signaling molecules in numerous developmental and physiological responses, playing a crucial role in regulating cell proliferation, apoptosis, and cellular senescence [20,21]. Generally, plant cells have mechanisms to maintain ROS homeostasis, such as antioxidant enzyme systems, including superoxide dismutases (SOD), peroxidases (POD), ascorbate peroxidases (APX), and glutathione transferases (GST), which activate the antioxidant cell protection system when faced with adverse stress [22]. Therefore, maintaining stable redox homeostasis is crucial for somatic embryogenesis.

It has been demonstrated that DNA methylation is closely linked to ROS. In a study by Poborilova et al., cytotoxic effects of juglone on tobacco BY-2 cells were investigated, and it was observed that treatment with juglone triggered ROS production, thereby inducing alterations in DNA methylation levels [23]. Furthermore, regarding fruit ripening, there is also an interaction between DNA methylation and ROS. Hypomethylation induced by 5-azaC was shown to effectively suppress ROS accumulation, particularly H_2_O_2_, in harvested strawberries, likely mediated by the activation of POD and CAT, contributing to delayed senescence [24]. A similar phenomenon was reported in salt-stress studies on kenaf (*Hibiscus cannabinus* L.). Pretreatment with 5-azaC was found to modulate methylation levels, which enhanced the activities of POD, SOD, and CAT, concurrently reducing ROS accumulation, increasing antioxidant capacity, and improving salt tolerance [25].

5-azaC is a demethylating agent, which has potential application in embryogenesis. This study specifically focused on investigating the effect of 5-azaC in the somatic embryogenesis of *Taxodium* hybrid ‘zhongshanshan’. By introducing varying concentrations and durations of 5-azaC treatment, this research shed light on the impact of 5-azaC on both EC and SE formation in *Taxodium* hybrid ‘zhongshanshan’.

## 2. Results

### 2.1. 5-azaC Inhibited EC Proliferation of Taxodium Hybrid ‘Zhongshanshan’

**Figure 1:**
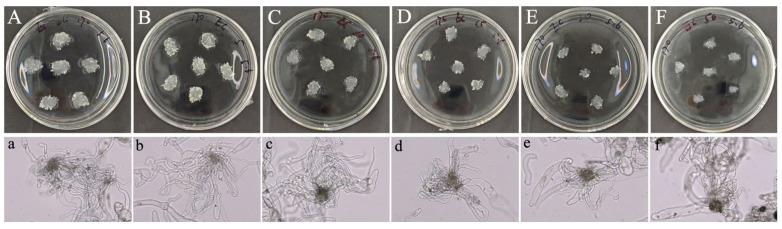
Morphological features of callus proliferation of *Taxodium* hybrid ‘zhongshanshan’ under different 5-azaC treatments. (**A**). callus from 0 μM 5-azaC (control); (**B**). callus from 5 μM 5-azaC; (**C**). callus from 10 μM 5-azaC; (**D**). callus from 15 μM 5-azaC; (**E**). callus from 30 μM 5-azaC; (**F**). callus from 50 μM 5-azaC; (**a**–**f**). the structure of callus under different 5-azaC treatments (0, 5, 10, 15, 30, 50 μM). The lowercase letters indicate the morphology of callus in the whole dish; the lowercase letters used in parenthesis indicate the microscopic structure of callus.

**Figure 2:**
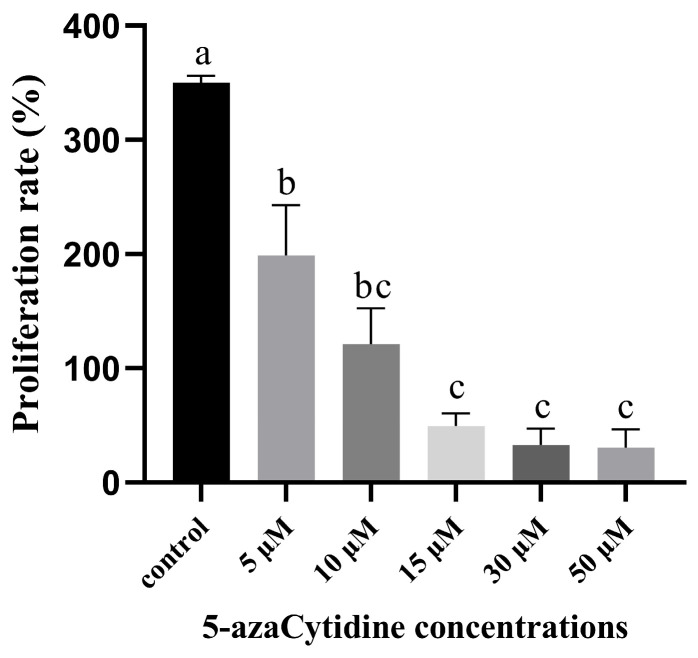
Effect of 5-azaC on the proliferation rate of EC in *Taxodium* hybrid zhongshanshan’. Statistical analysis of the proliferation rate presented as mean ± standard deviation. Mean values followed by standard deviation (vertical bars). Means followed by different letters are significantly different among treatments, according to the Tukey test (*p* < 0.05).

EC proliferation is the first step in somatic embryogenesis, as embryogenic cells within EC can differentiate into somatic embryos upon transfer to SE induction medium. In the EC proliferation study, compared to the control group, 5-azaC treatment significantly reduced EC proliferation. The results illustrated that the size of EC in the control treatment (Figure 1A) was considerably larger than that in the 5-azaC treatments (Figure 1B–F). Morphological assessments revealed similar white and transparent appearances, with a moist surface and no significant structural variances for both control (Figure 1a) and 5-azaC treatments (Figure 1b–f). Through statistical analysis, it was evident that increasing concentrations of 5-azaC, ranging from the control group to 50 μM, resulted in a significant decrease in the EC proliferation rate from an initial value of approximately 350% to around 30% (Figure 2). This reduction was statistically significant (*p* < 0.05). Therefore, it can be observed that the addition of 5-azaC inhibited the proliferation of EC.

### 2.2. 5-azaC Promoted Embryogenic Capacity of Taxodium Hybrid ‘Zhongshanshan’

**Figure 3:**
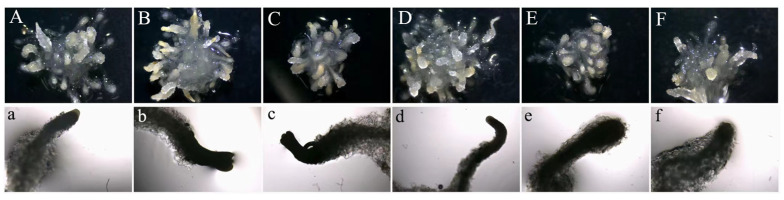
Morphological characteristics of SE of *Taxodium* hybrid ‘zhongshanshan’ under different 5-azaC treatments on the 40th day of development. (**A**–**F**). Morphology of a single callus with different concentrations of 5-azaC (0 (control), 5, 10, 15, 30, and 50 μM); (**a**,**d**–**f**) the columnar embryo of SE on the 40th day of development; (**b**,**c**). the mature cotyledonary embryo of SE on the 40th day of development.

**Figure 4:**
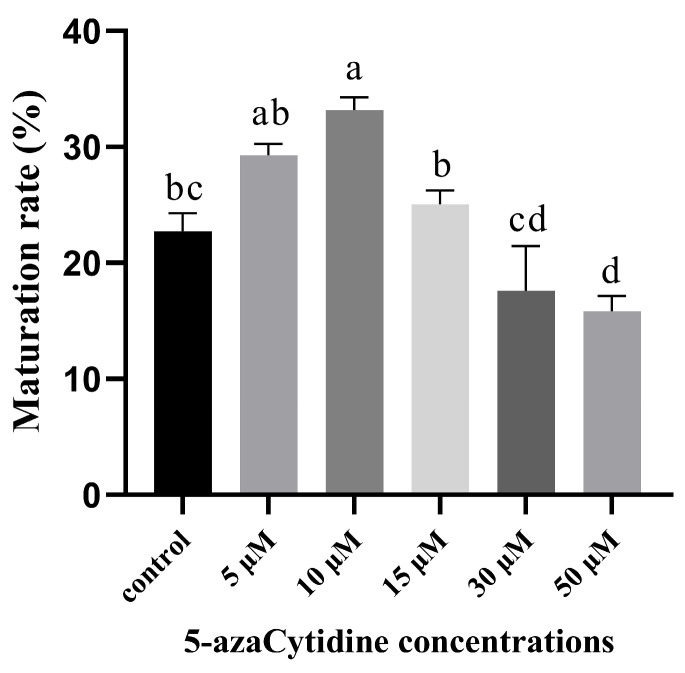
Effect of 5-azaC on the maturation rate of *Taxodium* hybrid zhongshanshan’. Statistical analysis of the maturation rate presented as mean ± standard deviation. Mean values followed by standard deviation (vertical bars). Means followed by different letter are significantly different among treatments, according to the Tukey test (*p* < 0.05).

On the 40th day of SE maturation stage without 5-azaC treatment (Figure 3A), and following treatments with different concentrations of 5-azaC (Figure 3B–F), we observed that lower concentrations of 5-azaC accelerated somatic embryogenesis, whereas higher concentrations of 5-azaC delayed the process of somatic embryogenesis. In the control treatment (Figure 3a), the first columnar embryo appeared on the 30th day, and the first mature cotyledonary embryo appeared on the 45th day (Table 1). After treatments with either 5 or 10 μM of 5-azaC (Figure 3b,c), the first mature cotyledonary embryo appeared on the 40th day, which was five days earlier compared to the control. The results demonstrated that treatments with concentrations of 5, 10, and 15 μM of 5-azaC increased both the number and maturation rates of mature cotyledonary embryos compared to the control (Figure 4). Among these treatments, 10 μM of 5-azaC had the highest maturation rate at 33.19%, which was 10.49% higher than that of the control (*p* < 0.05). Conversely, treatments with 30 and 50 μM 5-azaC reduced the number of mature cotyledonary embryos (Table S1), and the somatic embryos developed slowly. (Figure 3e,f). We also investigated 5-azaC in other embryogenic cell lines of *Taxodium* hybrid ‘zhongshanshan’, and the effects of 5-azaC on somatic embryo induction were the same as the results in this study (Figures S1 and S2).

### 2.3. The Best Time for Promoting Somatic Embryogenesis of 5-azaC Was in the Second Week

**Figure 5:**
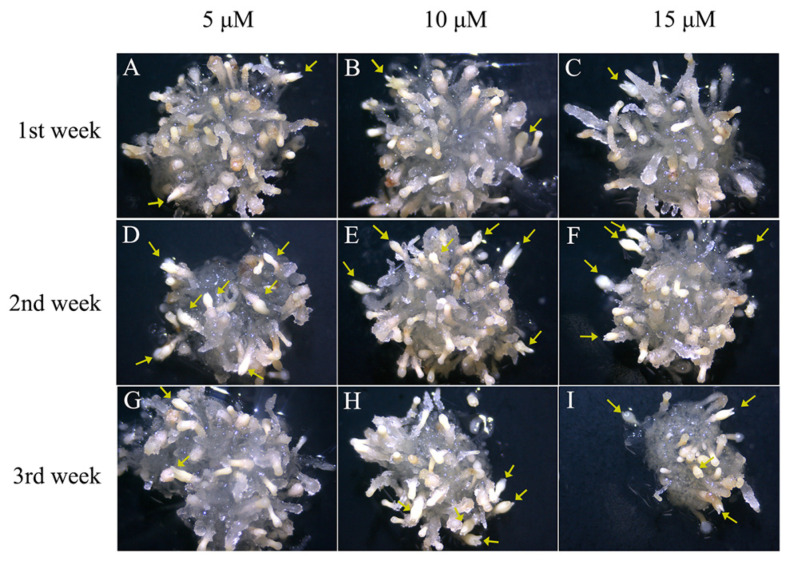
Mature cotyledonary embryos from a single callus were observed under the microscope at different action times of *Taxodium* hybrid zhongshanshan’ on the 60th day. Somatic embryos were cultured in an induction medium, and 5-azaC supplemented treatments with 5, 10, and 15 μM on the 1st, 2nd, and 3rd week of culture. (**A**–**C**). 5-azaC was added at concentrations of 5, 10, and 15 μM in the 1st week; (**D**–**F**). 5-azaC was added at concentrations of 5, 10, and 15 μM in the 2nd week; (**G**–**I**). 5-azaC was added at concentrations of 5, 10, and 15 μM in the 3rd week; yellow arrows indicate mature and maturing cotyledonary embryos.

**Figure 6:**
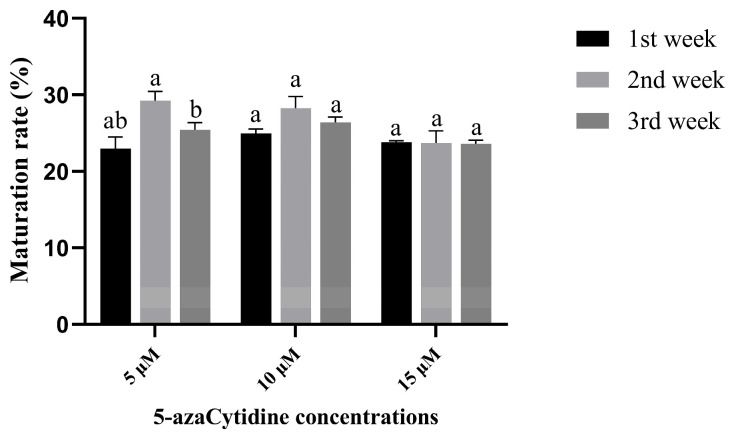
Effect of 5-azaC on the maturation rate under different action times of *Taxodium* hybrid zhongshanshan’. Statistical analysis of the maturation rate presented as mean ± standard deviation. Mean values followed by standard deviation (vertical bars). Means followed by different letters are significantly different among treatments, according to the Tukey test (*p* < 0.05).

The number of mature cotyledonary embryos obtained from treatments with 5-azaC at a concentration of 5 μM (Figure 5A) and 10 μM (Figure 5B) was higher compared to that at a concentration of 15 μM (Figure 5C) when 5-azaC was added in the first week of the SE process. The rate of somatic embryo maturation with the use of 5-azaC treatment increased when induction was performed in the second week as opposed to the first and third weeks. The cream-colored somatic embryos were evenly distributed and densely packed (Figure 5D–F). The maturation rate achieved by treating with 5-azaC in the third week (Figure 5G–I) was comparable to that observed after the first week of treatment. Furthermore, the highest maturation rate of 29.24% was attained with a 5-azaC concentration of 5 μM. There were no significant differences in the number of columnar embryos among treatments with different concentrations of 5-azaC during the first or second week of SE development. However, the number of columnar embryos treated with 5-azaC in the first and second weeks were significantly lower compared to those observed in the third week (Table S2). Compared to the first week of 5-azaC treatment, there was a noticeable increase in the number of mature cotyledonary embryos between the second and third weeks after 5-azaC treatments (Figure 6). This study demonstrated that low concentration treatments with 5-azaC have a positive effect on somatic embryo development in *Taxodium* hybrid ‘zhongshanshan’, particularly when added at a concentration of 5 μM in the second week of the SE process.

### 2.4. SE with 5-azaC Treatments Increased Plantlet Germination Rate

**Figure 7:**
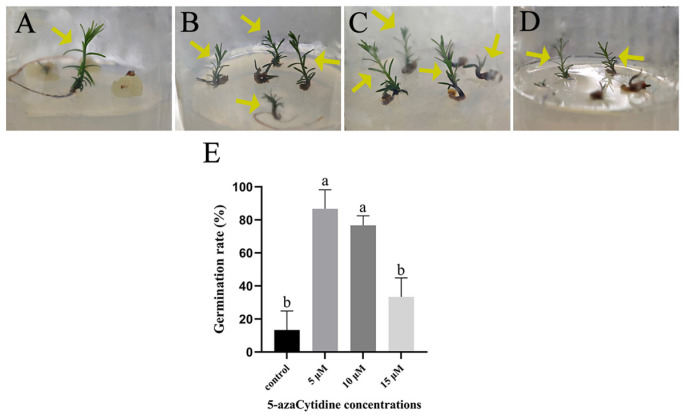
The germination of plantlet of *Taxodium* hybrid ‘zhongshanshan’. (**A**). The growth of plantlets was observed after a culture of 50 days onto germination medium supplemented with 0 μM 5-azaC (control); (**B**). the growth of plantlets was observed after a culture of 50 days onto germination medium supplemented with 5 μM 5-azaC; (**C**). the growth of plantlets was observed after a culture of 50 days onto germination medium supplemented with 10 μM 5-azaC; (**D**). the growth of plantlets was observed after a culture of 50 days onto germination medium supplemented with 15 μM 5-azaC; (**E**). the germination rate of *Taxodium* hybrid ‘zhongshanshan’ by 5-azaC. Means followed by different letters are significantly different among treatments, according to the Tukey test (*p* < 0.05). Yellow arrows indicate the *Taxodium* hybrid ‘zhongshanshan’ that has taken root and become a plantlet.

Fewer plantlets were observed after 50 days of development on WPM medium without 5-azaC treatment (Figure 7A). It was evident that the surfaces of somatic embryos that did not develop into plantlets were surrounded by yellow callus. In contrast, somatic embryos treated with 5-azaC did not exhibit this yellow callus formation, regardless of whether they had developed into plantlets or not. The number of plantlets increased significantly with a 5 μM 5-azaC treatment (Figure 7B). Compared to the control, the somatic embryos treated with 10 μM 5-azaC not only exhibited a higher plantlet germination rate but also demonstrated faster growth (Figure 7C). Although the growth rate of plantlets was slower with a 15 μM 5-azaC treatment, the number of plantlets was still greater than in the control group (Figure 7D). These findings suggest that low concentrations of 5-azaC (5, 10, and 15 μM) enhance both the plantlet germination rate and shorten the overall duration of Taxodium hybrid ‘zhongshanshan’ plantlet formation.

### 2.5. 5-azaC Affected Embryo Induction by Regulating Redox Homeostasis

**Figure 8:**
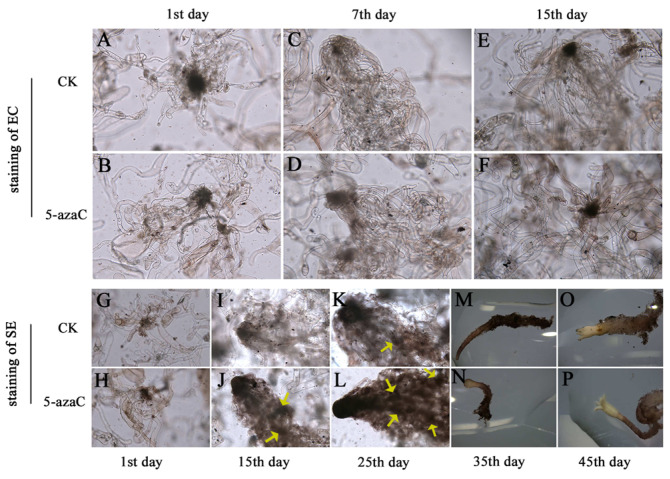
The microscopic observation of EC and SE with DAB staining. (**A**–**F**). Structure of EC stained with DAB, accumulation of H_2_O_2_ in cells by comparing the color changes of control and 50 μM 5-azaC treatment on the 1st, 7th, and 15th day; (**G**–**P**). structure of SE stained with DAB, accumulation of H2O2 in cells by comparing the color changes of control and 10 μM 5-azaC treatment on the 1st, 15th, 25th, 35th, and 45th day. Yellow arrows indicate the embryogenic suspensor mass exhibited reddish-brown staining with DAB.

**Figure 9:**
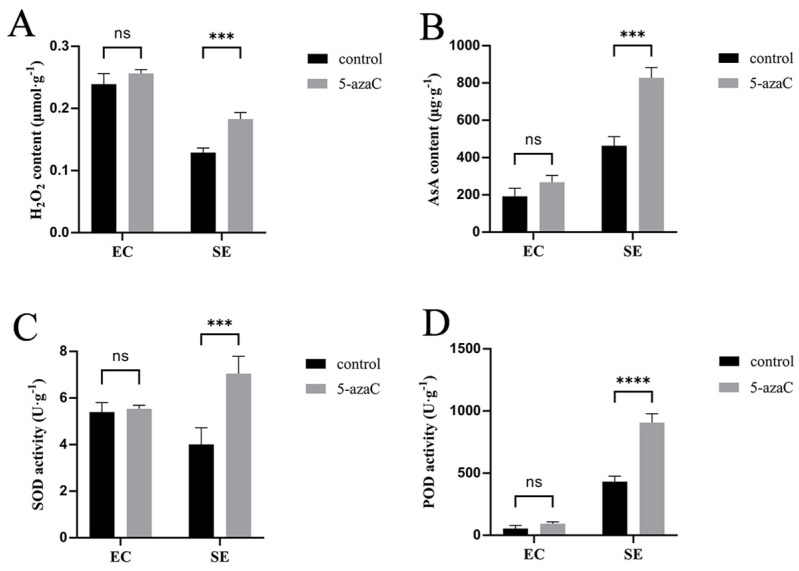
The content of ROS and AsA on the 60th day of maturation. (**A**,**B**) Changes in H_2_O_2_ and AsA content of EC and SE after 5-azaC treatments compared with control; (**C**,**D**) changes in SOD and POD enzyme activity of EC and SE after 5-azaC treatments compared with control. The asterisks indicate a statistically significant difference (*t* test, *** *p* < 0.001, **** *p* < 0.0001).

We performed histochemical localization and quantitative analysis of ROS in somatic embryos. After 3 h of DAB staining, both the control (Figure 8A) and the 50 μM 5-azaC treatment of EC (Figure 8B) appeared brown, with no noticeable difference in color on the 7th and 15th day (Figure 8C–F). The staining of SE on the first day, both for the control and 5-azaC treatment, appeared brown (Figure 8G,H). On the 15th day, the control staining of SE also appeared brown (Figure 8I), which is similar to the color of EC after staining. However, the SE treated with 10 μM 5-azaC appeared reddish-brown (Figure 8J) on the 15th day, which was darker than the color of the control after staining. The same color contrast was observed between the control and the 10 μM 5-azaC treatment on the 25th day (Figure 8K,L). By staining the cotyledonary embryos of control and 5-azaC-treated samples (Figure 8M–P), we found that there was no longer a significant change in color. Combining DAB staining with H_2_O_2_ content measurements revealed slightly higher H_2_O_2_ content in EC treated with 50 μM 5-azaC compared to the control, although not statistically significant. However, significantly higher H_2_O_2_ content was found in SE treated with 10 μM 5-azaC on the 60th day (Figure 9A). The activities of SOD, POD, and AsA followed similar patterns of variation (Figure 9B–D). Our results demonstrated a statistically significant difference in ROS levels between the 5-azaC treated group and the control.

## 3. Discussion

### 3.1. The Effect of 5-azaC on Somatic Embryogenesis Depends on Concentration and Action Time

Most studies that investigated the effects of 5-azaC on somatic embryos primarily focused on reducing tissue methylation levels [26,27], which demonstrated that high concentrations of 5-azaC hindered embryogenesis [28], whereas low concentrations promoted somatic embryo maturation [29]. The decrease in global DNA methylation levels could act as a pivotal regulatory mechanism, driving somatic cell de-differentiation and the re-establishment of totipotency. In this study, we evaluated the effect of lower concentrations (5, 10, 15, 30, and 50 μM) of 5-azaC on cotyledonary embryo maturation rates in *Taxodium* hybrid ‘zhongshanshan’. These results demonstrated that within the concentration range of 5–15 μM, 5-azaC exhibited a stimulatory effect on somatic embryogenesis of *Taxodium* hybrid ‘zhongshanshan’. Therefore, the impact of 5-azaC on somatic embryogenesis in *Taxodium* hybrid ‘zhongshanshan’ was dose-dependent.

Furthermore, we applied treatments with various concentrations during different periods and stages of somatic embryogenesis. Notably, it was observed that applying a low concentration in the second week increased the incidence of mature cotyledonary embryos. Similar results were observed in *Acca sellowiana*, where the application of 5-azaC at the initiation of somatic embryogenesis significantly enhanced embryo formation [30]. These findings differ from the research of Carneros et al. [31], who reported that the use of 5-azaC during pre-embryogenesis increased the proliferation of embryogenic masses but decreased the number of cotyledonary embryos compared to the control. When embryos were transferred to a medium without 5-azaC, the maturation rate of cotyledonary embryos was higher than that of the control, indicating that the addition of 5-azaC is effective. This is because 5-azaC contributed to enhancing embryogenic potential and the initiation of embryogenesis, and its action may occur during the pre-embryogenesis stage. This finding supported previous research, which indicated that applying 5-azaC during the pre-embryogenesis stage promoted somatic embryogenesis. However, there are a few examples where researchers have utilized or reported positive effects using the application of 5-azaC to promote organogenesis and somatic embryogenesis in plants [32], and most studies reported negative effects of the 5-azaC on somatic embryos [33,34].

In this study, 5-azaC was found to significantly enhance the plantlet germination rate of *Taxodium* hybrid ‘zhongshanshan’, suggesting a potential link between vegetative growth and DNA methylation. The same effect was observed in tissue culture of other plants species [35,36]. Additionally, mature cotyledonary embryos treated with 5-azaC demonstrated favorable anatomical structures, leading to improved germination and subsequent development into healthy plantlets [37,38].

Furthermore, studies indicated that the application of 5-azaC can enhance AsA content in fruits, thereby promoting tomato growth and development [39]. It was evident that 5-azaC could enhance the AsA content in plants, and this study demonstrated that mature cotyledonary embryos with elevated AsA levels have a higher probability of developing into healthy plantlets. This is attributed to the fact that AsA acts as a natural antioxidant, safeguarding cells against oxidative damage [40].

### 3.2. 5-azaC Promotes Somatic Embryogenesis by Regulating Redox Homeostasis

Prolonged tissue culture enhanced the level of methylation in calli [41], and some research has indicated that the process of re-differentiation from calli into plants was associated with an elevated level of methylation [42,43]. The cells would develop defense mechanisms to counteract the excessive damage caused by free radicals encountered during somatic embryogenesis. Therefore, redox homeostasis and methylation levels both have a major impact on plant somatic embryogenesis. Zeng et al. assessed the methylated cytosine and related ROS in the axillary buds, young callus, and aging callus in different parts of birch, confirming the dynamic relationship between methylated cytosine and redox homeostasis in cells [44]. Certain genes associated with induced methylation changes were related to adverse stress responses; therefore, DNA methylation was regarded as a molecular mechanism by which plants cope with stress conditions [45]. ROS accumulate significantly when plants are under adverse stress; in other words, there is a close relationship between DNA methylation levels and ROS. The process of somatic embryogenesis inevitably involves oxidative stress, and maintaining stable redox homeostasis can enhance cell differentiation, thereby promoting somatic embryogenesis.

In our study, we performed DAB staining of SE to explore the accumulation of H_2_O_2_, as there could be a connection between oxidative stress and H_2_O_2_ during plant tissue culture [46]. We found that SE from 5-azaC-treated samples were darker in color than the control after DAB staining, so we performed ROS assays on the SE. The results indicated that the H_2_O_2_ content was significantly increased in somatic embryos treated with 10 μM 5-azaC, and the SOD and POD activities were elevated. When plants are exposed to external stresses, such as high concentrations of H_2_O_2_ and other ROS, redox homeostasis is disrupted, leading to inhibition of normal cell growth [47]. Nevertheless, an appropriate concentration of H_2_O_2_ can promote somatic embryogenesis [48,49].

H_2_O_2_ is a form of ROS, and its excessive production can lead to oxidative stress. However, plant cells are equipped with a robust antioxidant enzyme system, including SOD, POD, and CAT, which can regulate the level of ROS to maintain redox homeostasis [50]. SOD is the first enzyme in the detoxifying process, which converts superoxide anion to H_2_O_2_ [51]. Guo et al. assayed the activity of POD in somatic cells of oak and concluded that POD facilitates the differentiation of somatic embryos, and the ability of embryogenesis was accompanied by an increase in the activity of POD [52]. This indicated that the increase in the levels of H_2_O_2_, SOD, and POD was beneficial to somatic embryogenesis. In the present study, the levels of H_2_O_2_, SOD, and POD were significantly increased after 5-azaC treatment, leading to an increase in the maturation rate of cotyledonary embryos and thus facilitating somatic embryogenesis. This further demonstrated that the application of low-concentration 5-azaC was associated with increased generation of ROS and enhanced activity of detox enzymes.

The research on DNA methylation inhibitors in plant growth regulation is still at the initial stage, and it is necessary to strengthen basic research and applied technology development. Determining safe and appropriate concentrations and durations of use for 5-azaC, minimizing or eliminating toxic effects, and improving application methods will require an in-depth exploration of its mechanisms of action on gene regulation.

## 4. Materials and Methods

### 4.1. Plant Materials

The EC of *Taxodium* hybrid ‘zhongshanshan’ was derived from the immature seeds which were collected from a cross of *T. distichum* (L.) Rich. (**♀**) and *T. mucronatum* Tenore (**♂**) in July 2022. The immature seeds were collected sterilized with ethanol and sodium hypochlorite, rinsed thoroughly, and dissected to inoculate embryos onto EC induction medium. The subculture medium comprised DCR medium supplemented with ascorbic acid (10 mg·L^−1^), casein hydrolysate (0.5 g·L^−1^), inositol (0.1 g·L^−1^), 6-benzylaminopurine (0.5 mg·L^−1^), 2,4-D (1 mg·L^−1^), glutamine (0.4 g·L^−1^), maltose (20 g·L^−1^), activated charcoal (2.5 g·L^−1^), phytagel (3 g·L^−1^).

### 4.2. The Impact of Different 5-azaC Concentrations on EC Proliferation

EC was maintained by regular subculturing every 15 days on EC subculture medium [53]. After 15 days of subculturing, ECs with vigorous growth were chosen and inoculated onto EC subculture medium containing 0, 5, 10, 15, 30, and 50 μM of 5-azaC (Aladdin, Article No. A100625, Shanghai, China), respectively. The pH of the medium was adjusted to a range of 5.2–5.3, then all the components in the medium were sterilized at 121 °C for 20 min. After sterilizing the medium at an appropriate temperature, sterile-filtered 5-azaC was added to the medium. All cultures were maintained in darkness at a constant temperature of 25 ± 2°C in an incubator. After a cultivation period of 15 days, the weight of the ECs was evaluated. Subsequently, a small sample of the EC was taken, gently placed on a microscope slide, mixed carefully with forceps after adding distilled water, covered with a coverslip, and the cellular morphology was observed under a microscope. Embryogenic calli were cultured on at least three replicate dishes per treatment; each dish was inoculated with six calli weighing approximately 0.2 g. The initial weight of the inoculum calli was recorded as W0 while the weight of calli after cultivation was measured as W1. The weight of proliferation of ECs was determined as W1 − W0, and the proliferation rate was calculated as (W1 − W0)/W0 × 100%.

### 4.3. The Impact of Different 5-azaC Concentrations on Embryo Development

After 15 days of subculturing, ECs with vigorous growth were chosen and inoculated onto maturation medium [54] containing 0, 5, 10, 15, 30, and 50 μM of 5-azaC, respectively. The maturation medium comprised DCR medium [55] supplemented with casein hydrolysate (0.5 g·L^−1^), inositol (0.1 g·L^−1^), polyethylene glycol (190 g·L^−1^) (YuanYe, Article No.V32174, Shanghai, China), glutamine (0.45 g·L^−1^), aspartic acid (0.2 g·L^−1^), proline (0.2 g·L^−1^), maltose (30 g·L^−1^), activated charcoal (2 g·L^−1^), phytagel (3 g·L^−1^), abscisic acid(ABA) (8 mg·L^−l^), and gibberellins (4 mg·L^−l^) (Shanghai YuanYe, Article No.S28506). The pH was adjusted to 6.0, then all the components in the medium (except ABA and GA) for filter-sterilization were sterilized at 121 °C for 20 min. After sterilizing the medium at an appropriate temperature, sterile-filtered ABA, GA, and 5-azaC were added to the medium. All the cultures were maintained in an incubator at a temperature of 25 ± 2 °C under dark conditions. Structures were observed every five days with a microscope. Embryogenic calli were cultured on at least three replicate dishes per treatment, with each dish inoculated with six calli, each weighing approximately 0.2 g. After a period of 60 days, the number of columnar embryos were denoted as N1 and the number of mature embryos denoted as N2; subsequently, the rate of somatic embryogenesis maturation was calculated using the formula: N2/(N1 + N2) × 100%.

### 4.4. Experiment on the Action Time of 5-azaC

After 15 days of subculturing, ECs with vigorous growth were chosen and inoculated onto DCR medium without 5-azaC. Subsequently, the ECs were transferred to a DCR medium containing 5, 10, and 15 μM of 5-azaC in the first, second, and third week of cultivation, respectively. The status of SE development was recorded through microscopic observation every 10 days. Embryogenic calli were cultured on at least three replicate dishes per treatment, with each dish inoculated with six calli, each weighing approximately 0.2 g. After a period of 60 days, the formation of SE was recorded. The number of columnar embryos was measured as N1, while the number of mature cotyledonary embryos was measured as N2. The rate of SE maturation was calculated as N2/(N1 + N2) × 100%.

### 4.5. The Impact of 5-azaC Treatments on the Growth of Taxodium Hybrid ‘Zhongshanshan’ Plantlets

The mature cotyledonary embryos obtained with the control were inoculated onto a germination medium, comprising WPM medium [56], sucrose 30 g·L^−1^, agar 8.4 g·L^−1^, and 5-azaC (0, 5, 10, and 15 μM). The pH of the medium was adjusted to a range of 5.7–5.8. After 30 days of culture, the dishes with mature cotyledonary embryos were placed for a week under low light conditions, and then the mature cotyledonary embryos were picked out and placed in flasks. All cultures were maintained in a light-incubated environment at a temperature of 25 ± 2 °C. Photographs of the plantlets were taken after 50 days of culture.

### 4.6. Histochemical Analysis of ROS and Determination of ROS

3,3′-Diaminobenzidine (DAB) (Coolaber, Article No. SL1805, Beijing, China) is a frequently utilized chromogenic substrate that reacts rapidly to produce a reddish-brown compound, catalyzed by peroxidase, to localize hydrogen peroxide in plants. The strength of the reddish-brown deposit indicates the degree of hydrogen peroxide accumulation [57].

Materials from each stage of EC (0 and 50 μM 5-azaC treatments) and SE (0 and 10 μM 5-azaC treatments) were transferred into centrifuge tubes, followed by the addition of 5 mL DAB solution. The tubes were then wrapped with aluminum foil and placed in a dark environment for 3 h, after which tissue staining was observed under a microscope.

Hydrogen peroxide (H_2_O_2_) concentration was determined with an H_2_O_2_ determination kit (Enzyme-linked, Article No. ml094984, Shanghai, China). A mortar was used to homogenize the samples after adding 1.0 mL of reagent 1 application extract to 1.0 g of calli. Afterwards, the homogenized solution was centrifuged at 8000× *g*/min for 10 min at 4 °C. The supernatant was collected, and the reagent was added, following the protocol specified in the kit. The absorbance was measured at 415 nm, and each treatment was performed in triplicate.

Superoxide dismutase (SOD) concentration was determined with SOD determination kit (Solarbio, Article No. BC0170, Beijing China). A mortar was used to homogenize the samples after adding 1.0 mL of reagent 1 application extract to 1.0 g of calli. Afterwards, the homogenized solution was centrifuged at 8000× *g*/min for 10 min at 4 °C. The supernatant was collected, and the reagent was added following the protocol specified in the kit, with the volume of sample liquid added to 490 μL. The absorbance was measured at 560 nm, and each treatment was performed in triplicate.

Peroxidase (POD) concentration was determined with a POD determination kit (Solarbio, Article No. BC0090, Beijing, China). A mortar was used to homogenize the samples after adding 1.0 mL of reagent 1 application extract to 1.0 g of calli. Afterwards, the homogenized solution was centrifuged at 8000× *g*/min for 10 min at 4 °C. The supernatant was collected, and the reagent was added following the protocol specified in the kit, with the volume of sample liquid added to 50 μL. The absorbance was measured at 470 nm, and each treatment was performed in triplicate.

Ascorbic acid (AsA) concentration was determined with an AsA determination kit (Jiancheng, Article No. A009-1-1, Nanjing, China). A mortar was used to homogenize the samples after adding 1.0 mL of reagent 1 application extract to 1.0 g of calli. Afterwards, the homogenized solution was centrifuged at 2500 rpm for 10 min at 4 °C. The supernatant was collected, and the reagent was added following the protocol specified in the kit. The absorbance was measured at 536 nm, and each treatment was performed in triplicate.

### 4.7. Statistical Analysis

The data of proliferation rate, maturation rate and germination rate were analyzed using one-way ANOVA in GraphPad Prism 9.5. Statistical analysis of the data, presented as means  ±  SE. The data of ROS content was analyzed using Tukey’s post hoc test in GraphPad Prism 9.5, the asterisks indicated a statistically significant difference (*t*-test, * *p* < 0.05, ** *p* < 0.01, *** *p* < 0.001, **** *p* < 0.0001).

## 5. Conclusions

Combined with the experimental findings of this study, exogenous addition of 5-azaC inhibited proliferation of EC and promoted somatic embryogenesis in *Taxodium* hybrid ‘zhongshanshan’. Upon assessing ROS levels in EC, we observed no alteration in redox state after 5-azaC treatment; thus, redox homeostasis does not account for the inhibition of EC proliferation by 5-azaC. However, a low concentration of 5-azaC positively affected the somatic embryogenesis of *Taxodium* hybrid ‘zhongshanshan’ at a specific time, as evidenced by changes in ROS levels during treatments with 5-azaC. In addition, we also found that low concentration of 5-azaC treatments also promoted the germination of plantlets. Therefore, we conclude that 5-azaC plays a role in regulating ROS levels during the embryogenesis process. Therefore, it is hypothesized that the application of low-concentration 5-azaC may lead to increased generation of ROS and enhanced activity of antioxidant enzymes, which could be responsible for the improved maturation and germination rates of somatic embryos in this study.

## Figures and Tables

**Table 1:** The first time of occurrence in columnar embryo and mature cotyledonary embryo of *Taxodium* hybrid zhongshanshan’ under different 5-azaC concentrations.

The First Time of Occurrence	Control	5 μM	10 μM	15 μM	30 μM	50 μM
Columnar embryo	30 d	30 d	30 d	35 d	35 d	40 d
Mature cotyledonary embryo	45 d	40 d	40 d	45 d	45 d	50 d

## Data Availability

All data supporting the findings of this study are included within the article. Further inquiries can be directed to the corresponding author.

## Supplementary Materials

The following supporting information can be downloaded at: https://www.mdpi.com/article/10.3390/plants14091354/s1, Figure S1: Morphological characteristics of SE of *Taxodium* hybrid ‘zhongshanshan’ with embryogenic cell line of Z17 under different 5-azaC treatments on the 45th day of development; Figure S2: Morphological characteristics of SE of *Taxodium* hybrid ‘zhongshanshan’ with embryogenic cell line of Z3 under different 5-azaC treatments on the 45th day of development; Table S1: The number of mature cotyledonary and columnar embryo with different concentrations of 5-azaC; Table S2: The number of mature cotyledonary and columnar embryo with different concentrations of 5-azaC at different time.

## Abbreviations

The following abbreviations are used in this manuscript:

5-azaC	5-azaCytidine
EC	Embryogenic cells
SE	Somatic embryo
WPM	Woody plant medium
ABA	Abscisic acid
GA	Gibberellins
ROS	Reactive oxygen species
H_2_O_2_	Hydrogen peroxide
SOD	Superoxide dismutase
POD	Peroxidase
AsA	Ascorbic acid
2,4-D	2,4-Dichlorophenoxyacetic acid
DAB	3,3′-Diaminobenzidine
