# Supplementary material for: 5-AzaCytidine Promotes Somatic Embryogenesis of Taxodium Hybrid ‘Zhongshanshan’ by Regulating Redox Homeostasis
Source: Plants (Basel). 2025 Apr 30;14(9):1354. doi: 10.3390/plants14091354 (PMC12073654; doi:10.3390/plants14091354)
Supplement: Supplementary file 1 [file plants-14-01354-s001.zip › plants-3569876-supplementary.pdf]

Supplementary data

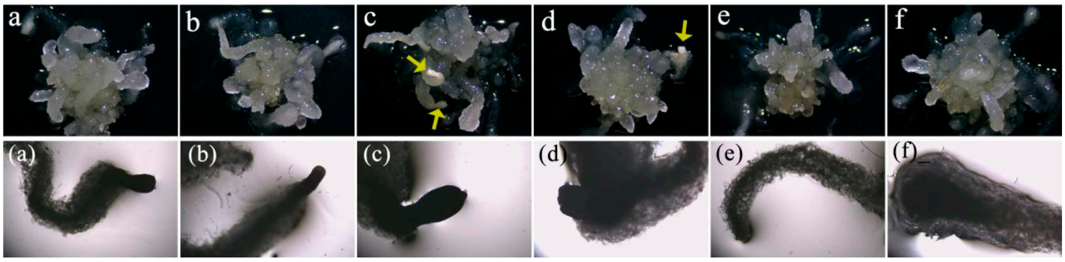

Figure S1. Morphological characteristics of SE of *Taxodium* hybrid 'zhongshanshan' with embryogenic cell line of Z17 under different 5-azaC treatments on the 45th day of development **a-f**, Microscopical morphology of a single callus with different concentrations of 5-azaC (0 (control), 5, 10, 15, 30 and 50 μM); **(a), (b), (e), (f),** The columnar embryo of SE on the 40th day of development; **(c), (d),** The immature cotyledonary embryo of SE on the 40th day of development.

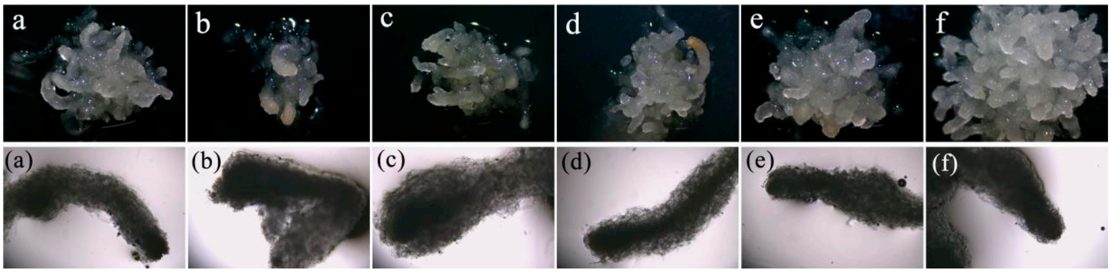

Figure S2. Morphological characteristics of SE of *Taxodium* hybrid 'zhongshanshan' with embryogenic cell line of Z3 under different 5-azaC treatments on the 45th day of development **a-f**, Microscopical morphology of a single callus with different concentrations of 5-azaC (0 (control), 5, 10, 15, 30 and 50 μM); **(a-f),** The columnar embryo of SE on the 40th day of development

Table S1. The number of mature cotyledonary and columnar embryo with different concentrations of 5-azaC.

Number	control	5 μM	10 μM	15 μM	30 μM	50 μM
No. of mature cotyledonary embryo	13.66±1.15	15.33±3.00	25.00±1.00	11.33±3.215	8.00±4.00	4.00±2.00
No. of columnar embryo	46.66±4.93	48.00±5.00	50.33±4.50	33.66±8.08	35.66±10.21	20.66±9.01

Table S2. The number of mature cotyledonary and columnar embryo with different concentrations of 5-azaC at different time

Action time	Number	5 μM	10 μM	15 μM
1st week	No. of mature cotyledonary embryo	22.33±1.15	27.66±2.08	19.66±1.52
	No. of mature columnar embryo	75.00±6.24	83.00±3.60	63.00±5.56
2nd week	No. of cotyledonary embryo	34.66±3.78	41.00±4.58	45.66±4.04
	No. of columnar embryo	83.66±4.72	104.66±17.04	146.66±3.055
3rd week	No. of mature cotyledonary embryo	45.33±4.04	42.00±7.81	28.33±7.02
	No. of columnar embryo	133.00±15.13	116.66±17.62	92.00±24.25
